# The Herbivore-Induced Plant Volatiles Methyl Salicylate and Menthol Positively affect Growth and Pathogenicity of Entomopathogenic Fungi

**DOI:** 10.1038/srep40494

**Published:** 2017-01-12

**Authors:** Yongwen Lin, Muhammad Qasim, Mubasher Hussain, Komivi Senyo Akutse, Pasco Bruce Avery, Chandra Kanta Dash, Liande Wang

**Affiliations:** 1 Plant Protection College, Fujian Agriculture and Forestry University, Fuzhou 350002, China; 2 State Key Laboratory of Ecological Pest Control for Fujian and Taiwan Crops, Fujian Agriculture and Forestry University, Fuzhou 350002, China; 3 Key Laboratory of Biopesticide and Chemical Biology, Ministry of Education, Fuzhou 350002, China; 4 Key Laboratory of Integrated Pest Management for Fujian-Taiwan Crops, Ministry of Agriculture, China, Fuzhou 350002, China; 5 Institute of Applied Ecology and Research Centre for Biodiversity and Eco-Safety, Fujian Agriculture and Forestry University, Fuzhou 350002, China; 6 University of Florida, Institute of Food and Agricultural Sciences, Indian River Research and Education Center, 2199 South Rock Road, Fort Pierce, FL 34945, USA

## Abstract

Some herbivore-induced-plant volatiles (HIPVs) compounds are vital for the functioning of an ecosystem, by triggering multi-trophic interactions for natural enemies, plants and herbivores. However, the effect of these chemicals, which play a crucial role in regulating the multi-trophic interactions between plant-herbivore-entomopathogenic fungi, is still unknown. To fill this scientific gap, we therefore investigated how these chemicals influence the entomopathogenic fungi growth and efficacy. In this study, *Lipaphis erysimi* induced *Arabidopsis thaliana* HIPVs were collected using headspace system and detected with GC-MS, and then analyzed the effects of these HIPVs chemicals on *Lecanicillium lecanii* strain V3450. We found that the HIPVs menthol and methyl salicylate at 1 and 10 nmol·ml^−1^ improved many performance aspects of the fungus, such as germination, sporulation, appressorial formation as well as its pathogenicity and virulence. These findings are not only important for understanding the multi-trophic interactions in an ecosystem, but also would contribute for developing new and easier procedures for conidial mass production as well as improve the pathogenicity and virulence of entomopathogenic fungi in biological pest management strategies.

Herbivore-induced-plant-volatiles (HIPVs) are emitted from plants after infestation by arthropods, and these volatiles are composed by many organic compounds which are involved in plant communication with natural enemies of the insect herbivores, neighboring plants, and different parts of the damaged plant. For instance, green leaf volatiles (GLVs) are comprised of C_6_ aldehydes, alcohols as well as esters, and terpenoids^1^,^2^.

It was well known that HIPVs play a significant role in attracting natural enemies of herbivores when plants become infested by herbivorous insects. Likewise, *Arabidopsis thaliana* (L) Heynh (Brassicales: Brassicaceae) attracted more parasitic wasp *Cotesia glomerata* (Linnaeus) (Hymenoptera: Braconidae), when the plant emitted a high amount of GLVs^3^. Recently, it has been reported that (Z)-3-hexenol, a unique compound of GLVs is the most important info-chemical for the natural enemy attraction^4^. A blend of six HIPVs compounds, such as beta-myrcene, n-octanal, and alpha-phellandrene, along with other host-nonspecific (E)-beta-ocimene, gamma-terpinene, and linalool played a role in the communication between plant and parasitic wasps *Aphidius ervi* Haliday (Hymenoptera: Aphidiidae) at a minimal quantity of 0.001 ng to 5 ng^5^. In addition, it also reported that predators respond to transgenic plant volatiles, like (E, E)-4, 8,12-trimethyltrideca-1, 3, 7, 11-tetraene, which is produced endogenously^6^. Furthermore, plant roots also emit HIPVs to attract natural enemies, such as; (E)-beta-caryophyllene. Furthermore, maize roots have been shown to attract entomopathogenic nematodes by using (E)-beta-caryophyllene after being attacked by the western corn rootworm, *Diabrotica virgifera* LeConte (Coleoptera: Chrysomelidae)^7^. It was also observed that when herbivorous insects oviposited on plants, they emitted electroantennographic-active compounds, such as (E)-4,8-dimethyl-1, 3, 7-nonatriene^8^, cis-3-hexen-1-ol, linalool and cis-alpha-bergamotene, which induced high egg feeding by generalist predators^9^.

Some studies have revealed the effect of HIPVs on pathogens, especially insect pathogen. Hountondji, *et al*.^10^ found that HIPVs promote the conidial growth of entomopathogenic fungus, *Neozygites tanajoae*. In another study, allyl isothiocyanate emitted by macerated wasabi plant (*Wasabia japonica* Matsumura) inhibited the growth of two entomopathogenic fungi *Beauveria bassiana* and *Isaria fumosorosea*^11^. Our previous study demonstrated that *A. thaliana* plant infested by *Lipaphis erysimi* Kaltenbach, emitted HIPVs compounds that promoted the performance of the entomopathogenic fungus (EPF) *Lecanicillium lecanii*, which was used for biocontrol of *L. erysimi*^12^. However, the role of each chemical component of the HIPVs induced by *L. erysimi*, is still unknown. It is therefore important to know how these chemicals influence the entomopathogenic fungi.

To fill in this gap, this study was designed and conducted at the College of Plant Protection, Fujian Agriculture and Forestry University, Fuzhou, China, where we collected HIPVs using headspace system and detected with GC-MS, and then treated the *L. lecanii* strain V3450 with similar synthetic chemical of HIPVs to analyze their biological function. *Arabidopsis thaliana* and *L. erysimi* were used to stimulate HIPVs production and collection. We therefore investigated and identified the components of these volatiles induced by *L. erysimi* in *A. thaliana* and synthetic chemicals from identified HIPVs were used to treat conidia of *L. lecanii*. The activity and effects of chemicals from HIPVs on the performance of *L. lecanii* were evaluated and the validity of various synthetic chemical’s concentrations to induce *L. lecanii* conidia was assessed.

## Results

### Headspace volatiles collected and detected by GC-MS

Nine HIPVs compounds notably limonene, 2-methyl-6-heptene, menthol, methyl salicylate, 1-octen-3-ol benzaldehyde, phenylacetaldehyde, decan-3-ol and terpineols were in total collected and identified from aphid-induced *A. thaliana*. Limonene and 2-methyl-6-heptene were detected in all the treatments (I, II, III, IV and V) i.e. independent of the different densities of *L. erysimi* apterous adults (1, 2, 5, 10 and 20) that infested *A. thaliana* plants, and with no significant differences (*F*_4, 10_ = 1.79, *P* = 0.23; *F*_2, 4_ = 3.99, *P* = 0.31) between treatments as regards to the quantities emitted (Table 1). Similarly, Menthol and 1-octen-3-ol were also collected from all treatments, but the quantities of Menthol produced in treatments III-V were significantly higher than those emitted in treatments I -II (*F*_4, 10_ = 50.89, *P* = 0.04); whereas the quantity of 1-octen-3-ol in treatment III was significantly higher than all the rest of the treatments (*F*_4, 10_ = 5.38, *P* = 0.009) (Table 1). Methyl salicylate was absent in treatment I but detected in all the other treatments II-V, and with the lowest quantity collected in treatment I compared to treatments II - V (*F*_3, 8_ = 30.74, *P* = 0.01) (Table 1). Likewise, benzaldehyde, phenylacetaldehyde and decan-3-ol were only detected in treatment III-V and completely absent in I and II. However, the benzaldehyde quantities collected from the three treatments were not significantly different (*F*_2, 6_ = 28.27, *P* = 0.1). The phenylacetaldehyde quantity was much higher in treatment V, compared to the other two treatments (*F*_2, 6_ = 84.81, *P* = 0.001), while the quantity of decan-3-ol emitted was significantly lower in treatment IV compared to treatments III and V (*F*_2, 6_ = 64.08, *P* = 0.03). In addition, Terpineols were only detected in treatment V (Table 1).

### Influence of aphid density on conidial germination and appressorial formation rate

The conidial germination and appressorial formation rate after exposure of *L. lecanii* strain V3450 to different concentrations of HIPVs induced by different densities’ number of aphids varied greatly after 12 h exposure (Table 2). For instance, the germination rate of conidia in treatments IV and V were significantly high (*F*_4, 4_ = 6.06; *P* < 0.05) compared to treatments I (control) (Table 2). Similar trend was observed for appressorial development where the highest rate of appressorial formation was obtained in treatment IV compared to other treatments (*F*_4, 4_ = 20.32; *P* < 0.0001). In addition, the rates have significantly increased (*P* < 0.0001) in treatment III and V compared with control (Table 2).

### Correlation analysis for HIPVs chemical compounds and conidial performance

The correlation between HIPVs chemicals and conidial performance of *L. lecanii* showed that the germination and appressorial formation rates were influenced by multiple HIPVs compounds as summarized in Table 3. For conidial germination, the correlation coefficient for menthol was higher than 0.5, indicating a significant correlation trend. Similarly, for appressorial formation, the correlation coefficients for menthol, methyl salicylate (MeSA), benzaldehyde and phenylacetaldehyde were all higher than 0.5, indicating a significantly strong correlation between these HIPVs compounds and the conidial performance (Table 3).

### Conidial performance after exposure to synthetic compounds

**Figure 1:**
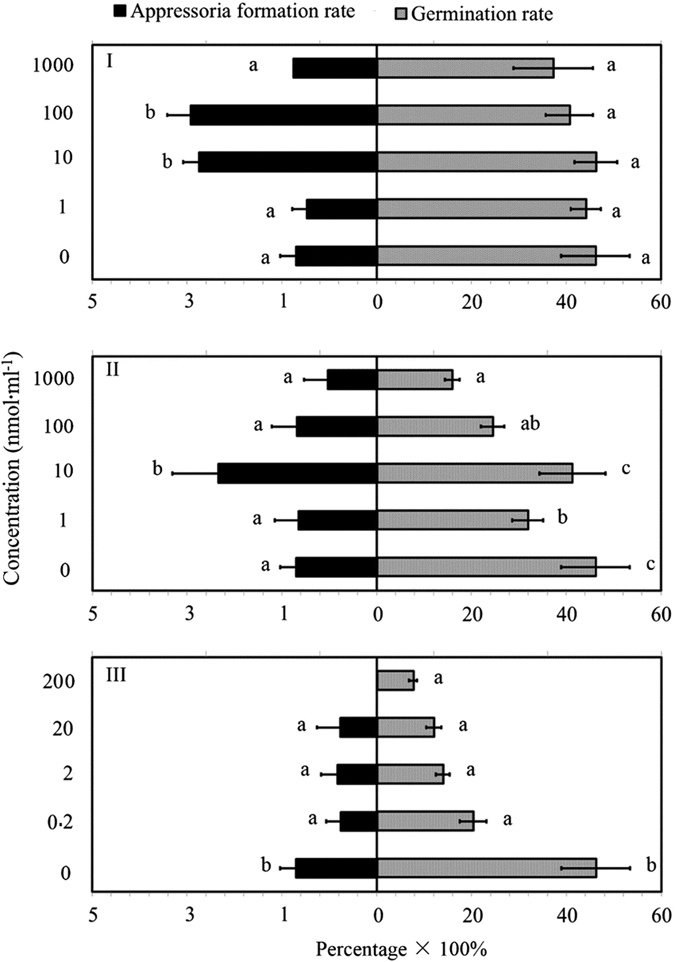
Germination and appressorial formation rate of *L. lecanii* treated with the different chemicals at different quantities for 12 h. Chemicals used in the test were (I) menthol, (II) methyl salicylate and (III) decan-3-ol. The number ‘0’ on the y-axis is the control treatment. The bars indicate mean value of germination and appressorial formation rate and the error bars indicate ± standard error (*n* = 5). The different letters above the error bars on each side indicates significance (Tukey’s test, *P* < 0.05).

*L. lecanii* were exposed to synthetic menthol, methyl salicylate, decan-3-ol, benzaldehyde and phenylacetaldehyde according to the results of GC-MS. When *L. lecanii* conidia were treated with menthol, no significant impact on their germination (*F*_4, 20_ = 0.41, *P* = 0.24; Fig. 1a) was observed, while conidial germination was significantly lower in the case of 1, 100 and 1000 nmol·ml^−1^ methyl salicylate application as compared to 10 nmol·ml^−1^ and the control (*F*_4, 20_ = 6.26, *P* = 0.03; Fig. 1b). Appressorial formation in the menthol treatment was boosted in the case of 10 and 100 nmol·ml^−1^, and same trend was observed with the 10 nmol·ml^−1^ methyl salicylate treatment (*F*_4, 20_ = 6.23, *P* = 0.022; *F*_4, 20_ = 2.03, *P* = 0.034; Fig. 1a,b). However, all decan-3-ol treatments had a negative impact on both the germination and appressorial formation of *L. lecanii* (*F*_4, 20_ = 17.89, *P* = 0.027; *F*_4, 20_ = 3.23, *P* = 0.033; Fig. 1c). In addition, conidia did not germinate in the treatment of benzaldehyde and phenylacetaldehyde.

### Hyphal growth

Impacts of synthetic chemicals on colony extension of *L. lecanii* after 15 days are summarized (Table 4). Growth speed of the fungal colony was faster when treated with 1 and 10 nmol·ml^−1^ menthol, and 1 nmol methyl salicylate (0.89, 1.09 and 0.92 cm·day^−1^, respectively), as compared to the control treatment (0 nmol·ml^−1^ treatment, 0.74 cm·day^−1^). Similarly, the diameter of the colony growth was also observed to be significantly larger after exposure to 1 and 10 nmol·ml^−1^ menthol (*F*_4, 20_ = 162.33, *P* = 0.0002), as well as 1 nmol·ml^−1^ methyl salicylate (*F*_4, 20_ = 34.83, *P* = 0.012) compared to the control, respectively (Table 4). However, the other chemical treatments (decan-3-ol, benzaldehyde, and phenylacetaldehyde) not only had a negative impact on the diameter of the fungal colony but also on the fungal growth speed (Table 4).

### Conidial production

**Figure 2:**
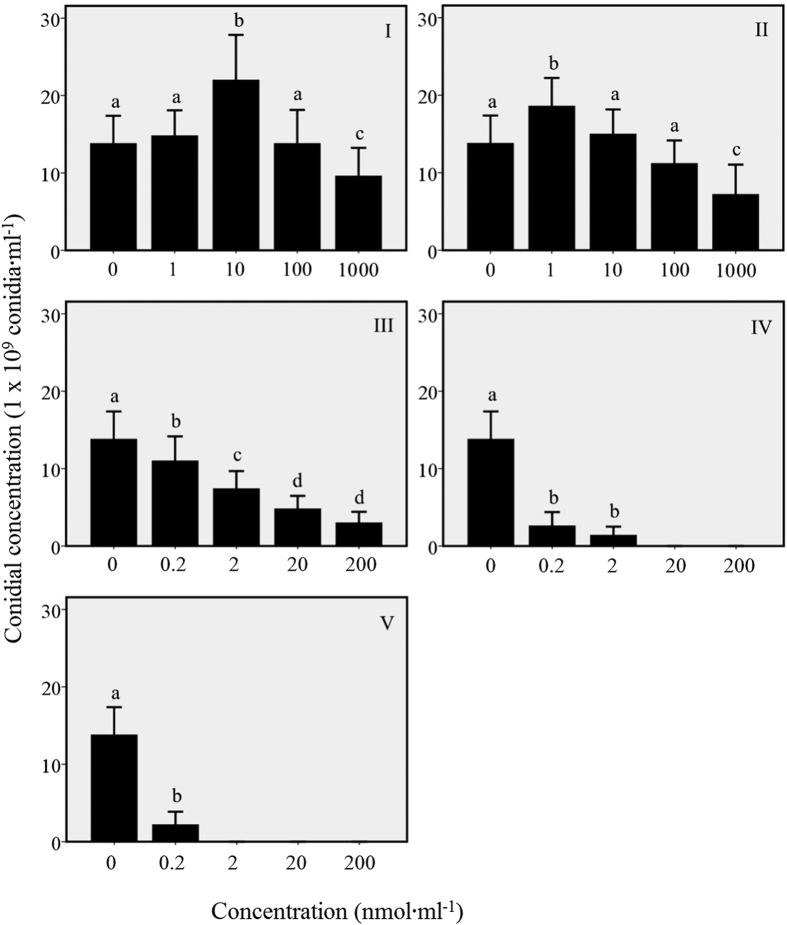
Sporulation of *L. lecanii* after exposure to compounds for 15 days. The chemicals which were used in the analysis are (I) menthol, (II) methyl salicylate, (III) decan-3-ol, (IV) benzaldehyde and (V) phenylacetaldehyde. The number ‘0’ on the x-axis is the control treatment. The bars indicate mean value of conidial concentration and error bars indicate a standard error (*n* = 5). The different letters above the error bars indicate significance (Tukey’s test, *P* < 0.05).

The effects of synthetic chemical compounds on the conidial production of *L. lecanii* after 15 days exposure are summarized in Fig. 2. Conidial production was enhanced by applications of 10 nmol·ml^−1^ menthol (*F*_4, 20_ = 22.57, *P* = 0.0003) and 1 nmol·ml^−1^ methyl salicylate (*F*_4, 20_ = 30.52, *P* = 0.0002), with significantly higher conidia yield than the control and other defined concentrations, respectively (Fig. 2a,b). However, conidial production was significantly inhibited under the tested concentrations in the treatments of decan-3-ol (*F*_4, 20_ = 59.63, *P* = 0.017), benzaldehyde (*F*_4, 20_ = 197.37, *P* = 0.0001) and phenylacetaldehyde (*F*_4, 20_ = 230.90, *P* = 0.0001) as compared to the controls (Fig. 2c–e). The controls therefore, yielded high conidia concentrations as compared to the decan-3-ol, benzaldehyde and phenylacetaldehyde treatments unlike in the cases of menthol and methyl salicylate (Fig. 2).

### Pathogenicity of Lecanicillium lecanii

The pathogenicity of *L. lecanii* to the adult aphids after being pre-treated with the chemicals was assessed (Table 5). *Lecanicillium lecanii* fungus exposed to menthol had a lowest LT_50_ (4.57 d) at the concentration of 10 nmol·ml^−1^ after 15 days post-exposure and with percent mortality significantly higher after 7 days compared to other chemical treatments as well as the control (*F*_4, 20_ = 19.36, *P* = 0.0021) (Table 5). A similar trend was observed for 1 nmol·ml^−1^ methyl salicylate treated fungus (*F*_4, 20_ = 24.34, *P* = 0.0001). For the other chemicals tested, the lethal time of the fungus as well as percent mortality was similar compared to the control (Table 5).

## Discussion

From the volatiles trapping, nine different compounds were emitted and identified as HIPVs when *A. thaliana* was infested with *L. erysimi*. Limonene, 2-methyl-6-heptene, menthol and 1-octen-3-ol were detected in all the aphid-induced *A. thaliana* plants. However, equal quantities of Limonene and 2-methyl-6-heptene were emitted independent of the aphid densities, while high quantities of menthol and 1-octen-3-ol were produced where aphid densities were increased (from 5 to 20 aphids). These results indicate that the quantities of some HIPVs (aphid-induced *A. thaliana*) compounds production depend on the densities of the herbivores. In addtition, our results indicated that, benzaldehyde, phenylacetaldehyde and decan-3-ol were produced only if the densities of aphids were between 5 and 20 adult aphids, while Terpineols were produced only when de density reached 20 aphids. This shows that some HIPVs could only be emitted if the pest population is high on the host plants as observed for Terpineols in aphid-induced *A. thaliana*. Furthermore, decan-3-ol, benzaldehyde, phenylacetaldehyde and salicylate acid were recorded in the headspace when *A. thaliana* was damaged by *L. erysimi*, and the quantity of menthol emitted from infested plants was significantly higher than control and the other HIPVs. Since insect herbivores are the key factor that induces HIPVs, it could be deduced from these results that the quantity and the composition of the emitted HIPVs might be influenced by the density of the pest population present at a particular time and stage of the host plants. Similar results were reported by Rodriguez-Saona, *et al*.^13^ through a gradual perennial shrub *Vaccinium corymbosum* infestation by gypsy moth caterpillars *Lymantria dispar* over time. The authors indicated that, the plant VOC emission rate also increased concomitantly as regards to the level of infestations. Our results also showed similar effects where the composition in terms of the number of HIPVs emitted from aphid-induced *A. thaliana* and their quantities increased with the subsequent increase in the number of aphids on the *A. thaliana* plant. In our previous finding and in this present study, HIPVs emitted from aphids-induced Arabidopsis increased performance of *L. lecanii*, and the rates of conidial germination as well as appressorial formation varied depending on the exposed aphid densities^12^. The results of this study showed that, decan-3-ol, benzaldehyde, phenylacetaldehyde, salicylate acid and menthol were considered as key synergetic compounds that enhanced conidial performance and consequently, improved the pathogenicity and virulence of *L. lecanii* strain V3450. Similar results were also reported by Lin, *et al*.^12^ using the same host plant and different aphid densities. In addition, we collected volatiles from 20 adult aphids in the glass jar of diagrammatic sketch of headspace collecting system, and analyzed the volatiles profile with GC-MS in a supplementary experiment (Supplementary experiment 3). The results showed that there were not menthol, methyl salicylate, decan-3-ol, benzaldehyde and phenylacetaldehyde in the headspace volatiles from aphids (Supplementary Fig. 1). These results suggested that the above mentioned chemical compounds (benzaldehyde, menthol, phenylacetaldehyde, decan-3-ol and methyl salicylate) found in the headspace volatiles were not emitted by *L. erysimi* but rather produced by the *L. erysimi*-induced Arabidopsis host plants.

When applied to *L. lecanii*, the five identified compounds produced different influences as regards to the level of fungal performance vis-a-vis aphid densities. Methyl salicylate and menthol did not provoke significant changes to the germination of the fungus, while decan-3-ol, benzaldehyde, phenylacetaldehyde inhibited the conidial germination. But when focusing on the appressorium formation, methyl salicylate (1 nmol·ml^−1^) and menthol (1 and 10 nmol·ml^−1^) showed their inducing function on *L. lecanii* performance, while the other compounds showed their inhibition function vis-a-vis to the fungus. This finding is significant because the formation of the appressoria is a precursor to the infection process of pathogenic fungi, which are swollen, dome-shaped cells^12^,^14^,^15^. These two compounds, menthol and methyl salicylate might therefore contribute to enhancing the pathogenicity and virulence of the entomopathogenic fungus *L. lecanii* with a synergetic effect. These results are similar to the previous research, which reported that salicylate acid indirectly promoted arbuscular mycorrhizal fungus in clover roots^16^. Spence, *et al*.^17^ also found that appressorial formation in *Magnaporthe oryzae* infecting rice was negatively impacted by hydrogen cyanide, a volatile produced by rhizospheric bacteria, had suppressed the rice blast infections.

In addition, when also applying the five identified compounds to the culture of *L. lecanii,* we found that methyl salicylate (10 nmol·ml^−1^) and menthol (1 and 1 nmol·ml^−1^) promoted hyphal growth and the toxicity of the entomopathogenic fungus increased significantly, while other compounds inhibited significantly the growth speed (sporulation) and consequently the pathogenicity of the fungus. Similarly, it was also found that methyl salicylate (10 nmol·ml^−1^) and menthol (10 nmol·ml^−1^) promoted sporulation significantly, while others inhibited significantly the fungal sporulation. These results indicated that methyl salicylate and menthol may increase or enhance the toxicity by up regulating the appressorial formation, hyphal growth and sporlulation of *L. lecanii.*

Entomopathogenic fungi (EPFs) are satisfactory candidates which have the potential to replace synthetic broad spectrum insecticides without having a major impact on the environment^18^,^19^. EPFs showed their potential and prospects of controlling insect vectors^20^,^21^,^22^, pests^23^,^24^,^25^,^26^,^27^ under laboratory and field conditions through inundative and autodissemination devices applications. Some of them, such as *Metarhizium anisopliae* and *Beauveria bassiana* had been used in the field with significant effects on their target pests^28^,^29^. EPFs infect insect pests and are not harmful to other participants of the particular agroecosystem. However, these EPFs encountered low pathogenicity, conidia viability and instability due to the effect of UVB irradiance, and as consequence, there are only few EPF products being used in the field^30^,^31^. In this regards, to overcome these challenges, many methods of molecular biology^32^,^33^, chemical biology^34^,^35^,^36^, and biophysics^37^,^38^ have been investigated to increase the use of EPFs under adverse field conditions and some studies look promising^39^. Therefore, our findings in this study are important for understanding the multi-trophic interactions in ecosystems as well as for developing new and easier means for increasing conidial production and improving the pathogenicity of EPFs. Further molecular studies are warranted to determine, which gene locus or loci is responsible for the production of these chemicals. Once identified, it might possibly be overexpressed as the gene segment to increase the efficacy of *L. lecanii* or promote its use in other EPFs.

## Methods

### Biological materials

*Arabidopsis thaliana* ecotype Colombia (Col-0) was used for all experiments. The plant was grown according to the methods of Hirao, *et al*.^40^ with slight modification. Instead of the 100-ml glass tubes used in reference, the 7-day-old seeding was transported into a 50 ml glass cup ( mm diameter, 8 mm length) (one seedling/cup), and incubated for 14 days in the growth chamber (23 °C, 70e80 mmol.m^−2^. s^−1^ fluorescent light, 16 h light/8 h dark, 65 RH). Plants visually healthy and not showed any damage for any other plant pest that could influence the volatile emissions were sampled or selected and used in the experiments.

*Lipaphis erysimi* were collected from cabbage fields at Fujian Agriculture and Forestry University (FAFU) campus and bred in mesh cages (50 cm × 50 cm × 50 cm) under laboratory conditions of 25 °C temperature, 75% RH, under a photoperiod of 16 L: 8D on *A. thaliana* for 5 generations prior to the experiments. Two-day-old apterous adults were collected from the established colony and used for the experiments.

The entomopathogenic fungus, *L. lecanii* strain V3450, was initially isolated from *Siphoninus phillyreae* Hasiday. This strain was obtained from China General Microbiological Culture Collection Center (CGMCC) in 2015, and cultured in the Laboratory of Insect Ecology in Fujian Agriculture and Forestry University. The fungus was identified according to morphological characteristics using the taxonomic keys for the genus *Lecanicillium*^41^,^42^.

### Headspace collection and volatiles analysis

**Figure 3:**
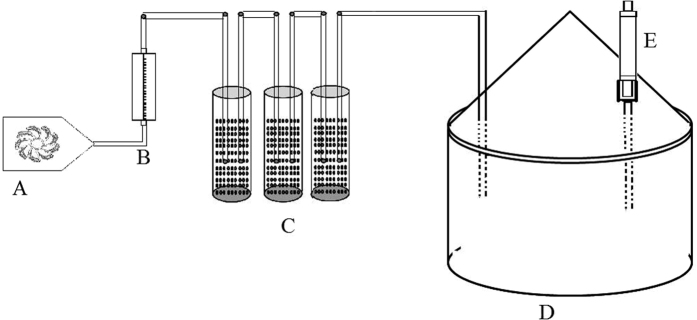
Diagrammatic sketch of headspace collecting system. (**A**) pump; (**B**) flower meter; (**C**) air-purify cup; (**D**) glass jar; (**E**) absorbing column.

The aphids-induced-Aradopsis plant volatiles were collected in the first 12 h using a headspace collecting system as shown in Fig. 3. According to the supplementary experiment 1, different densities of *L. erysimi* apterous adults (1, 2, 5, 10 and 20 which corresponded to five different defined treatments I, II, III, IV and V respectively) were released on *A. thaliana* for infestation for one hour. And then, each four infested plants with the same density of *L. erysimi* were transferred into a clean glass jar (12 cm diameter, 30 cm high). The air was blown into the jar at the rate of 300 ml·min^−1^ and flowed out of the headspace through a glass tube filled with 200 mg Tenax TA (60/80 mesh; Grace-Alltech, Deerfield, USA). After collection, the HIPVs were eluted into 1 ml methenyl trichloride and stored at −20 °C immediately. Four clean plants without aphid infestation were used as a control, and the experiment was repeated 3 times.

HIPVs were analyzed with a gas chromatograph (Agilent Technologies 7890B GC System)-mass spectrometer (Agilent Technologies 5977 A MSD) (GC-MS) via a HP-5 column (30 mm × 0.25 mm/1.0 μm film thickness, Agilent). The GC oven temperature was programmed from 40 °C (5-min hold), which was increased at a rate of 10 °C·min^−1^ regularly until it reached a temperature of 280 °C. The column effluent was ionized by electron impact ionization at 70 eV. Mass spectra were acquired by scanning from 35 to 350 m/z with the scanning rate of 5.38 scans·sec^−1^.

Compounds were identified by using the deconvolution software AMDIS (version 2.64, NIST, USA) in combination with NIST 05 and Wiley 7th edition spectral libraries and by comparing their retention indices with those from the literature. Quantities of headspace compounds were also compared with a standard sample quantity and by the detected peak area from GC-MS results.

### Influence of aphid density on conidial germination and appressorial formation

HIPVs were collected as described above based on the various aphid densities on the host plant. The air from the headspace was then allowed to flow into another jar (incubator jar) instead of absorbent column. There was 10 ml sterile water in the incubator jar to keep humidity for conidial germination. A concave slide was plastered onto the top of the incubator jar. Ten microliter of *L. lecanii* conidial suspension with a concentration of 1 × 10^8^ conidia·ml^−1^ was printed on the wall of the concave slide. Both conidial germination and appressorial formation of *L. lecanii* were determined after 12 h under a compound light microscope at 400X magnification for all the aphid density levels. The criterion used to assess conidial germination was that the length of germ tube > 50% the length of the conidia^12^. The treatment of 0 or no *L. lecanii* adults was used as control. Each test was conducted four times with 5 replicates.

### Efficiency of HIPVs compounds on fungal germination and appressorial formation

The results of GC-MS test and bioassay for conidial performance were analyzed by SPSS software 21.0 version to determine the correlation between single HIPVs chemical and germination rate or appressorial formation rate. Major compounds menthol, methyl salicylate, decan-3-ol, benzaldehyde, and phenylacetaldehyde identified in HIPVs of *A. thaliana* induced plant by the aphids revealed significantly high correlation with appressorial formation rate when different aphids’ densities were used as treatments (Table 3). Prior to the experiment, synthetic of similar identified compounds were dissolved in triethyl citrate (TEC) to achieve slow volatilization as described in Uefune, *et al*.^43^. A supplementary experiment was conducted to determine the experimental quantities of synthetic chemical (Supplementary experiment 2). All the synthetic chemicals previously listed above were purchased from Macklin Biochemical Co. Ltd. (Shanghai, China).

To assess germination and appressorial formation of conidia, a 2.5 μl conidial suspension (1 × 10^8^ conidia·ml^−1^) was prepared in Czapek’s liquid medium and placed onto the wall of a concave glass slide, and allowed to dry for 5 min^44^. A sterilized cotton roll was put into the jar of headspace collecting system (Fig. 3). One milliliter of each synthetic compound solution was applied on the cotton ball. The concentrations of menthol and methyl salicylate (MeSA) were 1, 10, 100 and 1000 nmol·ml^−1^ TEC, respectively; while the ones of decan-3-ol, benzaldehyde, and phenylacetaldehyde were 0.2, 2, 20 and 200 nmol·ml^−1^ TEC, respectively (Supplementary Table 2). The air from the headspace was then allowed to flow into another jar (incubator jar) instead of absorbent column as described above in the aphid density section. There was 10 ml sterile water in the incubator jar to keep humidity for conidial germination. A concave slides were plastered onto the top of the incubator jar. Ten microliter of *L. lecanii* conidial suspension with the concentration of 1 × 10^8^ conidia·ml^−1^ was printed on the wall of the concave slide. Both conidial germination and appressorial formation of *L. lecanii* were determined after 12 h under a compound light microscope at 400X magnification for all the defined concentrations. The criterion used to assess conidial germination was the same as describes above where the length of germ tube > 50% the length of the conidia^12^. Each test was conducted four times with 5 replicates per synthetic compound.

### Activity of synthetic compounds on fungal sporulation and growth

The fungus *L. lecanii* V3450 was cultured on Czapek’s solid medium and incubated at 25 °C, 75% RH and a photoperiod of 12:12 L:D for 14 days. Conidia were harvested by scraping the surface of sporulating cultures with an inoculating loop. Conidial suspension adjusted at the concentration of 1 × 10^8^ conidia·ml^−1^ was prepared with sterile water containing 0.05% Triton X-80^45^. Ten microliters of the fungal suspension were transferred to the center of round cellophane membranes (80 mm diameter) which covered the surface of the Czapek’s medium in a Petri dish (90 mm diameter). Sterilized tiny cotton wool ball was placed on the bottom of the Petri dish which was inoculated with *L. lecanii* V3450, and then amended with 1 ml of serial dilutions of each synthetic compound solution (1, 10, 100 and 1000 nmol·ml^−1^ TEC for menthol and MeSA; 0.2, 2, 20 and 200 nmol·ml^−1^ TEC for decan-3-ol, benzaldehyde, and phenylacetaldehyde). All the dishes were sealed tightly with Parafilm and kept in the incubator at 25 °C, at 75% RH, and with 12:12 L:D photoperiod. The range of dilutions used for the bioassay was based on the pre-experimental outputs mentioned above (Supplementary Table 2). Control petri dishes were treated with 0.1% Tween X-80, and each dilution was repeated 5 times for each synthetic compounds.

To access the growth rate, diameter of colony in each Petri dish was determined by vernier caliper every 24 h, and the diameters were measured for 14 days^46^.

To quantify conidial production, colonies and cellophane membranes were transferred into a 50 ml-centrifuge tube containing 10 ml sterile solution of 0.1% Tween-80 and filtered by three layers of sterile gauze to make conidial suspensions after 14-days incubation^47^. The concentration of suspensions was determined by vortexing for about 5 min to produce homogenous conidial suspensions. Conidial counts were then made using an improved Neubauer Hemacytometer to determine the right concentration of conidia.

### *In vivo* pathogenicity and virulence determination

The pathogenicity and virulence of *L. lecanii* to the adult aphids were assessed after the fungus was pre-treated with the HIPVs synthetic chemicals. Conidia from multi treated by synthetic chemicals was collected from previous experiment and made into conidial suspensions with distilled water respectively. Conidial suspensions were diluted to 5 × 10^7^ conidia·ml^−1^ with sterilized solution of 0.1% Tween-80 for infection bioassays. Fifty apterous adult *L. erysimi* per treatment were immersed in conidial suspension for 30 sec in a 50 ml beaker to inoculate them with fungal conidia. After inoculation, all *L. erysimi* were air dried for 5 min and transferred to cauliflower leaves (5 × 5 cm) with wetted cotton to keep them fresh^48^.

Each treatment in this experiment was repeated three times, while the control were treated with sterilized solution of 0.1% Tween X-80. The lethal time (LT_50_) and mortality were assessed where LT_50_ was analyzed by linear regression. The whole experiment was conducted in an illumination incubator under constant conditions of 25 °C, 75% RH, and 12:12 L:D photoperiod.

### Statistical analysis

Mortality of *L. erysimi* in treated groups was corrected by that observed in controlled conditions following the method described by Jones, *et al*.^49^. The median lethal time (LT_50_) was estimated using probit analysis in the statistical software SPSS 20.0. The means of compound quantities, fungal colony growth, conidial production, corrected mortality, germination rate and appressorial formation rate were compared by Tukey’s HSD test (α = 0.05) following an analysis of variance using the SPSS 20.0.

## Additional Information

**How to cite this article**: Lin, Y. *et al*. The Herbivore-Induced Plant Volatiles Methyl Salicylate and Menthol Positively affect Growth and Pathogenicity of Entomopathogenic Fungi. *Sci. Rep.* **7**, 40494; doi: 10.1038/srep40494 (2017).

**Publisher's note:** Springer Nature remains neutral with regard to jurisdictional claims in published maps and institutional affiliations.

## Supplementary Material

Supplementary Information

## Figures and Tables

**Table 1:** Quantities of major compounds in headspace volatiles released from *A. thaliana* infested by *L. erysimi.*

Compounds	Quantities ± SD (nmol)				
	I	II	III	IV	V
Limonene	3.10 ± 0.06a	3.80 ± 0.18a	3.23 ± 0.09a	3.05 ± 0.26a	2.82 ± 0.52a
2-Methyl-6-heptene	4.98 ± 0.13a	4.87 ± 0.23a	5.10 ± 0.34a	4.76 ± 0.29a	4.94 ± 0.39a
1-Octen-3-ol	1.44 ± 0.06a	1.33 ± 0.08ab	1.78 ± 0.15b	1.46 ± 0.14ab	1.57 ± 0.14ab
Menthol	0.41 ± 0.02a	0.47 ± 0.11a	3.26 ± 0.17b	3.68 ± 0.39b	2.17 ± 0.19c
Methyl salicylate	Nd	1.26 ± 0.05a	3.23 ± 0.26b	3.61 ± 0.35a	4.52 ± 0.56b
Benzaldehyde	Nd	nd	1.32 ± 0.25a	1.38 ± 1.05a	1.43 ± 0.12a
Phenylacetaldehyde	Nd	nd	1.01 ± 0.04a	1.07 ± 0.06a	1.46 ± 0.13b
Decan-3-ol	Nd	nd	1.06 ± 0.06a	0.67 ± 0.08b	1.29 ± 0.13a
Terpineols	Nd	nd	nd	nd	0.77 ± 0.06

Data show mean values of quantities ± SD (nmol) of main compounds in headspace which were collected for 12 h. There were 0, 2, 5, 10, 20 *L. erysimi* in treatment I, II, III, IV, V, respectively. Means followed by a different letter per compound emitted across each row per treatment are significantly different (Tukey’s HSD test, *P* < 0.05). ‘nd’ means not detected.

**Table 2:** The influence of headspace to conidial germination and appressorial rate.

Treatments	Insect density	Germination rate %		Appressorial formation rate %	
		mean value ± SE	confidence limited	mean value ± SE	confidence limited
I	0	54.86 ± 1.61a	57.59–65.79	5.06 ± 1.11a	3.68-0.64
II	2	59.33 ± 2.16ab	53.33–65.32	7.08 ± 1.87ab	4.76-9.41
III	5	61.69 ± 1.48ab	58.40–71.06	10.15–1.21b	8.65–11.66
IV	10	67.22 ± 2.08bc	61.44–73.01	13.97–1.20c	12.48–15.47
V	20	64.7 ± 2.28bc	50.38–59.33	8.49 ± 2.51b	5.37–11.61

Data shows germination and appressorial formation rate mean mortality ± SE of 5 replicates 12 h exposure to headspace at 25 °C. The HIPVs was emitted from Aradopsis was induced by different densities of aphids. Means followed by the different letter are significantly different (Tukey’s HSD test, P < 0.05).

**Table 3:** Analysis of correlation between the single HIPVs chemical and performance of conidia as influenced by HIPVs.

	Correlation analysis for germination rate and chemicals	Correlation analysis for appressorial formation rate and chemicals
Limonene	0.049	−0.265
2-Methyl-6-heptene	−0.144	−0.431
1-Octen-3-ol	0.173	0.285
Menthol	0.532	0.919
Methyl salicylate	−0.070	0.705
Benzaldehyde	0.162	0.772
Phenylacetaldehyde	−0.042	0.515
Decan-3-ol	−0.132	0.504
Terpineols	−0.782	−0.077

**Table 4:** The hyphal growth of *L. lecanii* when exposed to the different chemical compounds at different concentrations.

Series	Treatments		Fungal colony extension	
	Compounds	Quantities (nmol·ml^−1^)	Speed (cm·d^−1^)*	Diameter (cm)***
		0	0.74 ± 0.05	4.29 ± 0.21d
		1	0.89 ± 0.05	5.39 ± 0.24a
I	Menthol	10	1.09 ± 0.03	6.27 ± 0.11b
		100	0.71 ± 0.14	4.14 ± 0.09c
		1000	0.68 ± 0.04	4.19 ± 0.06c
		0	0.74 ± 0.05	4.29 ± 0.21d
		1	0.92 ± 0.04	5.37 ± 0.13a
II	Methyl salicylate	10	0.53 ± 0.02	3.17 ± 0.08b
		100	0.24 ± 0.03	1.58 ± 0.16c
		1000	0.14 ± 0.02	1.14 ± 0.05c
		0	0.74 ± 0.05	4.29 ± 0.21d
		1	0.62 ± 0.04	3.73 ± 0.12a
III	Decan-3-ol	10	0.58 ± 0.03	2.89 ± 0.07b
		100	0.25 ± 0.02	1.62 ± 0.08c
		1000	0.20 ± 0.01	1.37 ± 0.09c
		0	0.74 ± 0.05	4.29 ± 0.21b
IV	Benzaldehye	0.2	0.03 ± 0.02	1.09 ± 0.04a
		2	0.06 ± 0.01	0.77 ± 0.05a
V	Phenylacetaldehyde	0	0.74 ± 0.05	4.29 ± 0.21b
		0.2	0.05 ± 0.01	0.76 ± 0.04a

Data show mean value ± of the fungal colony of 5 replicates which cultured for 15 d. The number ‘0’ in all series are controlled treatments. Means followed by the different letter in a column are significantly different (Tukey’s HSD test, *P* < 0.05).

**Table 5:** Pathogenicity of *L. lecanii* pre-treated by the different chemical in different quantities.

Series	Treatments	Fungal pathogenicity to *L. erysimi*			
	Compounds	Quantities (nmol·ml^−1^)	LT_50_	95% Confidence limits (lower-upper)	Mortality (%)
		0	5.94	5.63–6.30	46.00 ± 2.67a
		1	4.94	4.68–5.23	49.33 ± 2.45a
I	Menthol	10	4.57	4.34–4.81	73.33 ± 4.35b
		100	6.04	5.69–6.42	46.00 ± 3.23a
		1000	5.87	5.53–6.29	38.00 ± 2.00a
		0	5.94	5.63–6.30	46.00 ± 2.67ab
		1	5.05	4.80–5.34	62.00 ± 2.71c
II	Methyl salicylate	10	6.30	5.91–6.80	50.0 0 ± 2.36ad
		100	7.08	6.55–7.79	37.32 ± 2.21be
		1000	7.60	6.95–8.53	32.00 ± 1.70e
		0	5.94	5.63–6.30	46.00 ± 2.67a
		1	6.05	5.71–6.46	36.67 ± 3.33a
III	Decan-3-ol	10	6.62	6.24–7.12	35.33 ± 4.55a
		100	7.05	6.54–7.73	40.66 ± 3.71a
		1000	7.52	6.93–8.36	34.66 ± 3.42a
		0	5.94	5.63–6.30	46.00 ± 2.67a
IV	Benzaldehye	0.2	7.32	6.79–8.04	42.67 ± 4.14a
		2	7.90	7.22–8.88	34.66 ± 2.67a
V	Phenylacetaldehyde	0	5.94	5.63–6.30	46.00 ± 2.67a
		0.2	7.80	7.13–8.77	38.67 ± 3.43a

Data shows LT_50_ value and percent mean mortality ± SE of 3 replicates 15 d post-exposure at 25 °C. LT_50_ were analyzed by linear regression. The number ‘0’ in the quantities column represent control treatments. Means followed by the different letter are significantly different (Tukey’s HSD test, *P* < 0.05).
