# Supplementary material for: The Herbivore-Induced Plant Volatiles Methyl Salicylate and Menthol Positively affect Growth and Pathogenicity of Entomopathogenic Fungi
Source: Sci Rep. 2017 Jan 12;7:40494. doi: 10.1038/srep40494 (PMC5227919; doi:10.1038/srep40494)
Supplement: Supplementary Information [file srep40494-s1.doc]

**The Herbivore-Induced Plant Volatiles Methyl Salicylate and Menthol Positively affect Growth and Pathogenicity of Entomopathogenic Fungi**

**Yongwen Lin^1, 2, 3^, Muhammad Qasim^1, 2, 3^, Mubasher Hussain^1, 2, 3^, Komivi Senyo Akutse^1 ,2,3^, Pasco B. Avery^4^, Chandra K. Dash^1, 2, 3^, Liande Wang^1, 2, 3*^**

Supplementary Information

**Supplementary experiment 1**

To determine and confirm the suitable density of the aphid *Lipaphis erysimi* pressure on the *Arabidopsis* *thaliana* in this pre-experiment, the following trial was conducted. At first, 0, 1, 10, 20, 30, 40 and 50 adult aphids were transferred onto *A.* *thaliana* plants, respectively. However, it was hard to transfer more than 50 individual onto the small plant due to the high aphid density pressure. Herbivore-induced-plant volatiles (HIPVs) were collected with diagrammatic sketch of headspace collecting system based on the various aphid densities (0, 1, 10, 20, 30, 40 and 50) on the host plant. The air from the headspace was then allowed to flow into another jar (incubator jar) instead of absorbent column. There was 10 ml sterile water in the incubator jar to keep humidity for conidial germination. A concave slide was plastered onto the top of the incubator jar. Ten microliter of *L. lecanii* conidial suspension with a concentration of 1 x 10^8^ conidia·ml^-1^ was printed on the wall of the concave slide. Both conidial germination and appressorial formation of *L. lecanii* were determined after 12 h under a compound light microscope at 400X magnification for all the studied aphid density levels. The criterion used to assess conidial germination was that the length of germ tube > 50% the length of the conidia. The treatment of 0 or no *L. lecanii* adults was used as control. Each test was conducted four times with 5 replicates. The results showed and suggested that the density of the insect should not be more than 20 (Supplementary Table 1), so we arranged the serial of insect density as 0, 2, 5, 10 and 20.

Supplementary Table 1. The influence of headspace to conidial germination and appressorial rate

| Insect density  (aphids/per plant) | Germination rate % | | Appressorial formation rate % | |
| --- | --- | --- | --- | --- |
|  | mean value±SE | confidence limited | mean value±SE | confidence limited |
| 0 | 54.61±4.01a | 44.66∼64.56 | 5.08±0.35ad | 4.22∼5.94 |
| 1 | 57.53±2.54a | 51.23∼63.83 | 6.63±0.35b | 5.76∼7.50 |
| 10 | 67.48±2.08b | 62.31∼72.66 | 1.37±0.03c | 1.29∼1.44 |
| 20 | 64.41±2.54b | 58.1∼70.720 | 8.24±0.79de | 6.28∼10.21 |
| 30 | 57.31±1.44a | 53.72∼60.89 | 6.09±0.22de | 5.55∼6.64 |
| 40 | 56.89±1.78a | 52.47∼61.30 | 5.65±0.12e | 5.36∼5.94 |
| 50 | 54.53±2.05a | 49.43∼59.63 | 5.44±0.31e | 4.68∼6.20 |

Data shows germination and appressorial formation rate mean mortality ± SE of 5 replicates 12h exposure to headspace at 25°C. The HIPVs was emitted from Aradopsis was induced by different densities of aphids. Means followed by the different letter are significantly different (Tukey’s HSD test, P < 0.05).

**Supplementary experiment 2**

In this pre-experiment, five chemicals (menthol, methyl salicylate, decan-3-ol, benzaldehyde, and phenylacetaldehyde) were applied on the cotton roll with the quantities of 1, 10, 100 and 1000nmol (chemicals were dissolved and diluted with TEC). The cotton roll then put into the jar of headspace collecting instrument and Tanax A was used to be absorbent in this experiment. Clean air was forced into the jar at at the rate of 300 ml·min^-1^ with pump. GC-MS was used to detect the quantities with the same plotting parameters as before.

From the results of this pre-experiment (Supplementary Table 2), we chose 1, 10, 100 and 1000 nmol per ml TEC for menthol and methyl salicylate; 0.2, 2, 20 and 200 nmol per ml TEC for decan-3-ol, benzaldehyde, and phenylacetaldehyde. We made this choice not only to simulate the natural situation, but also detect the impact trend of HIPVs chemicals to conidial.

Supplementary table 2. Quantities of chemical emitted from cotton roll in different treatments in 12 hours

| chemical | Quantities of HIPVs emitted from 10-aphids-infested plant treatment (nmol)* | Quantities of chemical emitted from cotton roll (nmol) | | | |
| --- | --- | --- | --- | --- | --- |
|  |  | I | II | III | IV |
| Menthol | 3.68 ± 0.39 | 0.45±0.03 | 3.70±0.19 | 27.73±0.82 | 180.50±0.65 |
| methyl salicylate | 3.61 ± 0.35 | 0.71±0.01 | 4.41±0.11 | 38.65±0.77 | 236.59±1.88 |
| Benzaldehyde | 1.38 ± 1.05 | 0.76±0.02 | 4.61±0.14 | 44.22±1.27 | 306.65±2.71 |
| Phenylacetaldehyde | 1.07± 0.06 | 0.69±0.02 | 4.25±0.07 | 45.75±1.13 | 314.64±0.68 |
| decan-3-ol | 0.67 ± 0.08 | 0.25±0.01 | 2.19±0.17 | 17.43±0.62 | 109.89±0.71 |

*The data was copied from table 1 in main article of this research. In treatment I, II, III, IV, chemicals were applied on the cotton roll at the quantities of 1, 10, 100 and 1000 nmol, respectively.

**Supplementary experiment 3**

In the supplementary experiment, we put 20 adult aphids without *Arabidopsis* *thaliana* plant in a glass jar, then collected headspace volatiles from 20 adult aphids with diagrammatic sketch of headspace collecting system, and analyzed the volatiles profile with GC-MS. The results showed that, benzaldehyde (7.666 min), menthol (11.311 min), phenylacetaldehyde (11.792 min), decan-3-ol (16.803 min) and methyl salicylate (18.696 min) were found in the headspace volatiles from *L. erysimi*-induced Arabidopsis (Supplementary Fig 1-B), but we could not find these above mentioned chemicals in headspace volatiles from *L.erysimi* alone (Supplementary Fig 1-A). These results suggested that benzaldehyde, menthol, phenylacetaldehyde, decan-3-ol and methyl salicylate found in the headspace volatiles were not emitted by  *L. erysimi* but rather produced by the *L. erysimi*-induced Arabidopsis host plants.


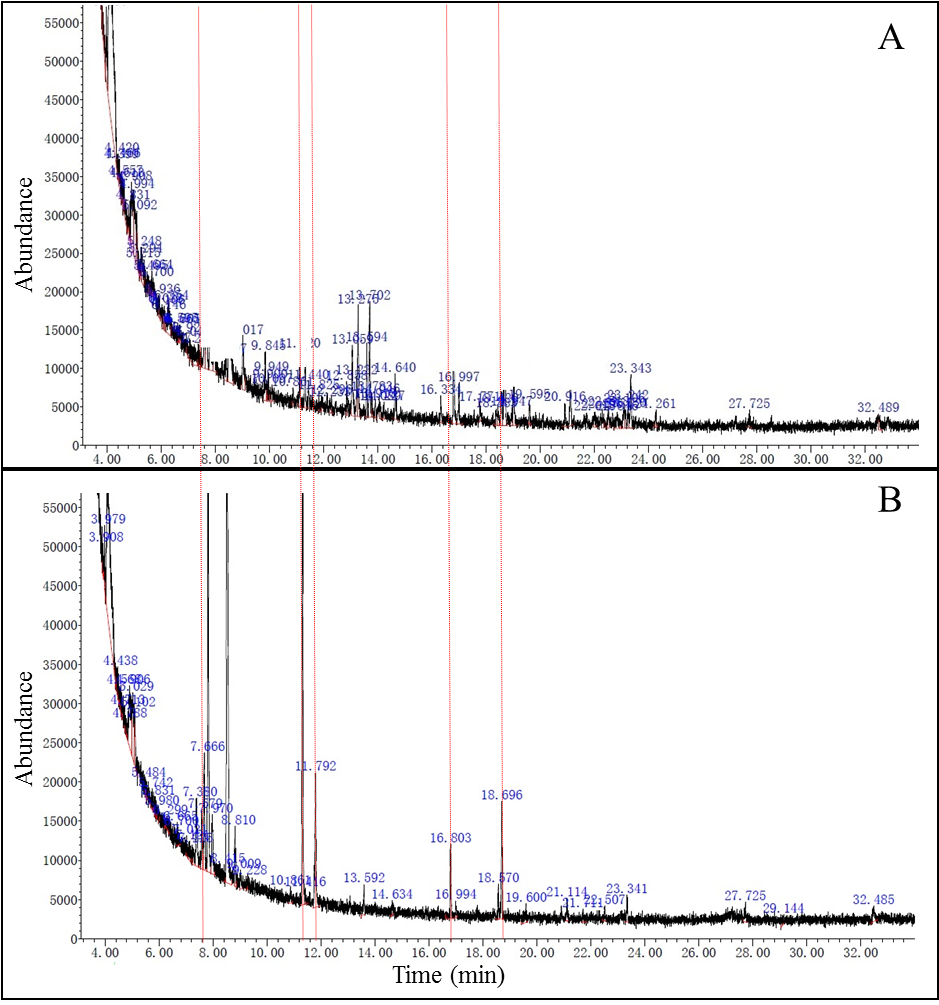


Supplementary Fig 1. Total ion chromatograms of headspace volatile compounds from *Lipaphis erysimi* and *L. erysimi*-induced Arabidopsis host plant. A, ion chromatograms of headspace volatile compounds from *L. erysimi*; B, ion chromatograms of headspace volatiles from *L. erysimi*-induced Arabidopsis host plants.
